# The Impact of Dietary Intervention in Obese Children on Asthma Prevention and Control

**DOI:** 10.3390/nu14204322

**Published:** 2022-10-15

**Authors:** Hanna Sikorska-Szaflik, Joanna Połomska, Barbara Sozańska

**Affiliations:** 1st Department and Clinic of Paediatrics, Allergology and Cardiology Wrocław Medical University, ul. Chałubińskiego 2-2a, 50-368 Wrocław, Poland

**Keywords:** asthma, obesity, allergy, prevention

## Abstract

The prevalence of both asthma and obesity in the pediatric population is steadily increasing, and even the obese–asthma phenotypes are postulated. Obese children with asthma experience more asthma symptoms, more frequent exacerbations, and worse response to treatment; they also report a lower quality of life compared with lean asthmatics. Some of the etiological factors for asthma and obesity may overlap. Perhaps asthma and obesity share a common genetic and immunologic origin. Diet is a compelling modifiable factor in obesity and asthma prevention and control, although the relationship between these two diseases is certainly multifactorial. In this article, we analyze the impact of dietary intervention and weight loss in obese children on asthma prevention and control.

## 1. Introduction

Obesity was commonly considered to be a cosmetic defect for many years. Now it is well-known that excessive body weight is the cause (sometimes the main and sometimes coexisting) of many diseases, e.g., endocrine, cardiovascular, and respiratory, including asthma. The pathogenesis of obesity is certainly multifactorial. In children, excess body weight is predominantly associated with improper eating habits and insufficient physical activity.

As there is a significant increase in the prevalence of both obesity and asthma in the pediatric population, a growing number of studies also concerning the relationship of these two diseases are being conducted. Observational studies showed the association between obesity and asthma; the association that is most often postulated is the influence of obesity on the occurrence of asthma [1,2], and there are also reports indicating the bidirectional link [3]. The basis for such a relationship would be genetic, immunological, and environmental factors (including diet and lifestyle).

Obese children with asthma experience more asthma symptoms, more frequent exacerbations, and worse response to treatment; they also report a lower quality of life compared with lean asthmatics [4]. The question is if the introduction of nutritional education leading to improving the diet and weight reduction may be a chance to prevent asthma in children. In this article, we want to analyze the impact of dietary intervention and weight loss in obese children on asthma prevention and control.

## 2. Influence of Obesity on the Occurrence and Course of Asthma

Asthma is a heterogeneous condition, and due to the differences in pathophysiological background and response to treatment, it is divided into several endotypes, although the clinical symptoms are similar in various ones. There is also a division of asthma into phenotypes which have different clinical manifestations and are defined as observable individual features resulting from genetic and environmental factors. Several asthma phenotypes have been described. Holguin et al. postulated the presence of early onset and late-onset obese–asthma phenotypes [5]. Early onset asthma, according to the authors, usually begins under the age of 12 and affects boys and girls equally. It is usually atopic in origin—patients have elevated levels of total immunoglobulin E. The airway infiltration is eosinophilic, and the Th2 biomarkers are elevated. Usually, in this group of patients, asthma occurs first and then obesity, complicating the course of the disease. Patients are characterized by a reduction in spirometric parameters, significant respiratory tract hyperresponsiveness, and more severe symptoms. In contrast, late-onset asthma is usually diagnosed in patients over 12 years of age, more often in women. Atopy is not observed. The airway infiltration is neutrophilic, and the Th2 biomarkers are low. Most often, obesity precedes the onset of asthma in that phenotype. Airway hyperresponsiveness and airway obstruction are also lower, and patients report less severe disease symptoms [5,6,7]. In early onset asthma, as it is eosinophilic and complicated with obesity, the treatment plan should consist of anti-inflammatory medication and weight loss [5], whereas in the late-onset asthma, weight loss should be a priority to gain better asthma control [8]. Peters et al. suggested the existence of the obese–asthma syndrome, which would include the phenotypes of asthma described above, and also another one associated with increased response to environmental pollutants [9]. Scott et al. demonstrated another phenotype—neutrophilic obese–asthma, but it was only found in adult women [10]. The variety of phenotypes of obese–asthma is considerable and not entirely clear. Understanding, for example, of the early onset of asthma may be complicated by the influence of obesity in pregnant women on the incidence of asthma in their children. It has been shown that obesity in pregnant women increases the risk of asthma in their child by 21–31%, and exactly that an increase in body mass index (BMI) by 1 kg/m^2^ increases the risk of early onset asthma by 2–3% [11]. 

The timing of the weight gain plays a role in a child’s level of risk for developing asthma. Children who gain an excessive amount of weight over the first 6 years of life, especially during infancy, are at an even higher risk for asthma than children who gain weight later in childhood [12]. 

Based on a Canadian study in 10-and-11-year-olds there is a relationship indicating that, with an increase in BMI by 1 kg/m^2^, the incidence of asthma increases by 6%, both in girls and boys. This result was not influenced by the gender of the respondents, the presence of allergies, or socioeconomic factors [13]. Gilliland et al., in an observational study, which initially included healthy children, showed that the risk of asthma was higher in overweight or obese children, and that this effect was greater in boys and in non-allergic children [14]. Similarly, a meta-analysis of studies related to BMI and asthma confirmed that overweight or obesity increases the risk of asthma by 1.64 and 1.92 times, respectively, compared with normal weight patients. However, most of the studies showed that this relationship is more frequent in girls, or there is no difference between the sexes [15].

Obesity may cause the incidence of diseases imitating asthmatic-like symptoms: gastroesophageal reflux and obstructive sleep apnea. Children may experience shortness of breath or chest pain, which can be misdiagnosed as asthma. A proper diet (thus avoiding obesity) will minimize such a diagnostic problem [6,11].

Obesity in children leads to a decreased response to inhaled glucocorticoids (GCSs). In obese subjects, inflammation is characterized by neutrophilia (less influenced by GCS) rather than eosinophilia. At the same time, due to the general inflammation in the body and the action of pro-inflammatory cytokines, obese people have real resistance to GCSs, and this is associated with altered GCS receptor quality. No change in the effect of short-acting β-agonists (SABAs) was observed in obese patients [16,17].

## 3. The Etiological Connection between Obesity and Asthma

### 3.1. Genetic

Several genes that can cause both diseases are being investigated. Candidate genes include, but are not limited to, PRKCA, LEP, ADRB2, ACE, and TNF. The genome-wide association study (GWAS), involving more than 23,000 children and adults, raised the possibility that the DENND1B gene could be common to the pathogenesis of both diseases. However, subsequent studies do not provide conclusive results [18]. In a more recent GWAS, the genetic relationship between obesity and asthma was studied, and the result revealed the relationship between obesity and late-onset and non-atopic asthma only [19]. Studies conducted on twins prove the genetic relationship between asthma and obesity, but they also emphasize the complexity of this relationship and the influence of many factors, apart from genetic ones [20,21].

### 3.2. Immunologic

Obesity may be regarded as a chronic systemic inflammation with the participation of factors referred to as adipokines, which are standard hormones produced by adipose tissue; leptin; and adiponectin. Moreover, Tumor Necrosis Factor alpha (TNF-α), Interleukin-6 (IL-6), and the chemokines IL-8 and Monocyte Chemoattractant Protein-1 (MCP-1) also complement proteins and other acute-phase proteins [22]. An increase in the expression of protein genes associated with inflammation has been shown in obese subjects [22]. According to the reports, adipokines are released into the blood and, after reaching the respiratory tract, may affect their hyperresponsiveness. Compared to lean patients, obese ones have elevated blood levels of pro-inflammatory factors, which decrease along with a decrease in BMI [23]. 

Leptin, a hormone derived from adipose tissue, is significantly increased in children with asthma [24] and in obese people [25]. A relationship of leptin with asthma severity, reduction in spirometric parameters, and increased BMI has been shown [26]. Leptin production by adipocytes can be induced by infectious and inflammatory stimuli, including TNF-α and IL-1β [27]. Leptin also has a pro-inflammatory effect by stimulating the cytokine secretion [27]. Due to its influence on lymphocytes, the production of Th1 cytokines is increased, and the proliferation of Treg decreases. It may be one of the mechanisms that leads to asthma. Higher leptin levels in obese adolescents inversely correlate with forced expiratory volume in one second (FEV1), forced vital capacity (FVC), and FEV1/FVC [24]. However, leptin-related relationships can also vary by gender. For instance, Quek et al. have shown that girls with asthma have higher levels of leptin [28]; however, Guler et al. studied school-aged children with asthma and found a significant difference in serum leptin levels between healthy children and those with asthma. This difference, however, only concerned boys [29].

The concentration of adiponectin in adipose tissue and plasma decreases with increasing body weight and increases again after the patient loses weight [24]. Adiponectin administered exogenously in mice protects them against obesity-related diseases, e.g., type 2 diabetes, thus emphasizing the important function of this protein [23]. The role of adiponectin can be both pro-inflammatory and anti-inflammatory. For example, adiponectin reduces TNF-α-induced NF-κB activation in endothelial cells and reduces LPS-produced TNF-α production in macrophages. It has also been shown that adiponectin increases the expression of some anti-inflammatory factors, including IL-10 and the endogenous IL-1 receptor antagonist [23]. At the same time, adiponectin causes a dose-dependent increase in IL-6 release from fibroblasts and macrophages. Adiponectin receptors are expressed on airway epithelial cells, and adiponectin induces the release of IL-8 in these cells [30]. Thus, the exact role of adiponectin may depend on the nature of the stimulus and the cells it acts on.

Leptin and adiponectin are also associated with exercise-induced changes in lung function [31]. Baek et al. demonstrated that the level of leptin was higher and that adiponectin was lower in obese asthmatics compared with lean ones, and the concentration of these hormones was significantly correlated with bronchial hyperresponsiveness after exercise. In obese children, bronchospasm is more easily induced by exercise than by methacholine provocation [31].

Higher levels of oxidative stress markers are observed in patients with asthma. Likewise, when the amount of adipose tissue is increased, especially visceral fat, the level of oxidative stress is higher. It has not been unequivocally proven whether obesity can affect the onset and course of asthma through the excessive production and action of reactive oxygen species, for example, by damaging the epithelium of the respiratory tract [9].

The metabolic disorders accompanying obesity also affect the course of asthma. Hyperglycemia and hyperinsulinemia can lead to bronchial hyperresponsiveness and narrowing of the bronchi through epithelial damage and proliferation of airway smooth muscles. Metabolic syndrome and insulin resistance are associated with decreased lung function in adolescents, both with asthma and healthy adolescents. Obesity-related pro-inflammatory cytokines such as IL-6 may play a key role in the relationship between metabolic syndrome, lung function, and the severity of asthma [9].

### 3.3. Mechanical

The mechanical effect of obesity on asthma may also be important. Excessive body fat may put pressure on and constrict the chest; moreover, fat accumulated in the abdomen makes it harder for the diaphragm to move down, leading to increased work of breathing [32]. Functional residual capacity and expiratory reserve volume are reduced in obese patients, regardless of asthma [22]. Unlike obese adults with decreased FEV1 and FVC, obese children are characterized by decreased FEV1/FVC [33]. It may be related to airway dysanapsis, which is observed not only in obese patients. It refers to the situation when the growth of the lung tissue and the lungs’ volume is greater, and at the same time, the enlargement of the caliber of the airways is inadequate, i.e., smaller [34].

### 3.4. Others

Other factors can also have an effect on the coexistence of asthma and obesity, e.g., low birth weight, which is connected with asthma and obesity. Likewise, antibiotic therapy in early childhood, by changing the gut microbiome, can affect the occurrence of both diseases in childhood [7].

## 4. Effect of Dietary Interventions/Weight Loss on the Occurrence and Course of Asthma

As the association between asthma and obesity is multifactorial, maybe it is possible to reduce the incidence of asthma and improve its course in obese patients by physical activity, weight loss, and dietary intervention.

### 4.1. Physical Activity

Eijkemans et al., in 2011, created a systematic review describing studies regarding physical activity and the risk of asthma. Researchers found that physical exercise may be a potential protective factor against developing asthma. At the same time, the authors emphasized possible limitations of their review—a greater number of analyzed studies were cross-sectional in design rather than longitudinal (better for causality assessment), and also the impact of other confounding factors (e.g., exposure to tobacco smoke) cannot be eliminated [35].

### 4.2. Weight Loss (Physical Activity and Diet)

In patients with already diagnosed asthma, it has been shown that physical exercise to reduce body weight with or without caloric restriction diet or at least normocaloric diet improves asthma control. Jensen et al. used a 10-week diet in adolescents with asthma, resulting in a decrease in their BMI and an improvement in ACQ (Asthma Control Questionnaire). At the same time, the authors did not register any improvement in spirometric parameters or any decrease in fractional exhaled nitric oxide (FeNO), leptin, eosinophils, or neutrophils [36].

Obese adolescents suffering from asthma achieved weight reduction, as well as improvement in asthma-related quality of life, after 28 weeks of a normocaloric diet. Moreover, in comparison with the control group, patients had fewer asthma exacerbations (less SABA intake and less nighttime wakefulness) during the study period [37]. In another research study, obese adolescents with asthma were encouraged to go on a diet and to exercise. A decrease in leptin and an increase in adiponectin concentration in their serum were reported [38]. Willeboordse et al., in an 18-month study of obese children with asthma or with a high risk of asthma, revealed improvement in asthma control, lung parameters, and asthma quality of life (PAQLQ) after intervention of weight reduction. Changes were seen in both the intervention and control group, but improvement in FVC% predicted, asthma control, and asthma quality of life were more visible in children from the intervention group [39].

Similarly, in adult patients with asthma, weight loss improves asthma control. However, this relationship occurs under certain conditions. In the Breathe Easier through Weight Loss Lifestyle (BE WELL) Intervention study, a large 12-month project with 330 participants, Ma. et al. suggest that weight loss of more than 10% of the baseline value has an impact on asthma control (expressed as statistically significant improvement in ACQ) [40]. Moreover, Scott et al. found that weight loss in overweight adult asthmatics must be at least 5–10% to improve asthma control, lung function, and quality of life [41]. Weight loss (caused by diet, exercise, or both) by more than 10% was connected with greater improvement in FVC, but not with better ACQ or Asthma Quality of Life Questionnaire (AQLQ). On the other hand, weight loss of less than 5% did not improve FVC, but improved ACQ and AQLQ. According to Johnson et al., 5% weight loss may be enough for obese asthmatics to gain clinically significant improvement in asthma control and quality of life [42]. In children, no relationship has been found so far that a specific percentage of body weight should be lost to improve asthma control. Interestingly, patients whose weight loss was caused by diet and exercise showed better asthma-control results when compared with patients whose weight loss was only due to diet. Thus, the importance of exercise in obese asthmatics was emphasized [43]. A prospective study was conducted among obese patients with severe asthma. The study group was on a weight-reduction program (encouraged to lose weight by eating less caloric meals and taking medication), and after 6 months, the level of asthma control from using the ACQ was obtained. During the study, patients who managed to lose weight had less asthma symptoms, fewer visits to the emergency room, and less rescue medication use. Moreover, an increase in forced vital capacity was shown in that group; however, no changes in markers of airway inflammation or bronchial reactivity were shown compared to the control group [44].

In studies conducted on adults, medications are often used to help them lose weight, and patients scheduled for bariatric surgery are also followed. The results of such studies show that asthma is better controlled after weight loss [8,45]. Sparse studies show similar improvements in pulmonary parameters in extremely obese adolescents after weight loss through bariatric surgery [46,47].

### 4.3. Diet

Losing weight can improve asthma control in obese. It is likely that dietary patterns implemented in early life may contribute to the onset of asthma and obesity.

Breastfeeding was shown to be relevant in protection against the development of asthma and obesity [48,49]. Not only the fact of breastfeeding but also the duration of it is crucial in asthma and obesity protection [50].

One of the causes of obesity might be the use of a Western diet based on a large number of calories, sweets, fats, and processed foods and a small amount of fruit, vegetables, fiber, and micronutrients. Moreover, for asthma, a Western diet is regarded as a possible risk factor. Consuming more fat and less fiber was shown to cause a higher eosinophil rate in the airways and worse spirometric parameters, i.e., lower FEV1 and higher leptin levels [51]. Such a diet may promote a pro-inflammatory state due to the lack of a sufficient amount of antioxidants and, thus, increased susceptibility to oxidative stress. In addition, increased consumption of saturated fatty acids by activating TLR4 receptors leads to the activation of the inflammatory cascade of NF-κB [52]. In contrast, a Mediterranean diet consisting of fruits, vegetables, nuts, fish, unsaturated fatty acids, and antioxidants has an anti-inflammatory effect. Examining Spanish children, Garcia-Marcos et al. revealed that obesity was a risk factor of current severe asthma in girls aged 6 and 7, and the Mediterranean diet may have a protective effect on it [53]. In French school-aged children from a PARIS birth cohort, high adherence to a Mediterranean diet was connected with lower asthma morbidity and sensitization and also with higher FEV1 and FVC in patients [54]. However, there are also some studies not confirming the positive relation between a Mediterranean diet and asthma prevention [55]. Sexton et al. studying asthmatic adults on a Mediterranean diet showed that diet intervention improved patients’ asthma related quality of life, but this improvement was not statistically significant [56]. In a pilot study, Bseikri et al. showed that improving a diet by a specially designed nutrient-and-fiber-dense bar may improve lung function in poorly controlled asthmatic adolescents with obesity, even without losing weight or making special changes in lifestyle. However, it was only in non-eosinophilic asthma (with low FeNO measures in patients) [57].

### 4.4. Vitamin D

Obese and asthmatic children often suffer from vitamin D deficiency [58,59]. It is not clearly established whether vitamin D supplementation could help prevent asthma and obesity; however vitamin D’s multidirectional effect explains its possible impact on children with obese–asthma. Studies suggest that obese children require higher doses of vitamin D in comparison with lean ones and also that losing weight can increase vitamin D concentration [60]. Vitamin D immunomodulation of anti-inflammatory processes in the airways has been regarded as one of the possible ways of influencing the course of asthma [61]. This effect during vitamin D deficiency is less effective. The causes of vitamin D deficiency in the obese are different—maybe it is because of the fact that vitamin D, being soluble in fats (present in excess in the obese), has lower blood concentration, which leads to its weakened effectivity [62]. Another possibility is an effect of high leptin and IL-6 in the obese, as they have an influence on VDR receptors and lead to lower 25(OH)D synthesis [63]. The other important fact is that obese asthmatics exercise less and spend less time outdoors, thus preventing UV-dependent vitamin D skin synthesis. Epidemiological studies show increased frequency of respiratory infections in obese people. Vitamin D deficiency is regarded as one of the causes [64]. Respiratory tract infections, on the other hand, are one of the factors that exacerbate asthma. The multidirectional influence of vitamin D on asthma and obesity is significant.

### 4.5. Other Factors

In obese adolescent asthmatics, Lang et al. supplemented omega-3 fatty acid (n3PUFA) through 6 months and observed lung function and biomarkers in comparison to the control group not receiving n3PUFA. Their outcomes show no improvement in spirometry, Asthma Control Test, asthma exacerbations, and urgent-care visits for asthma after the study period [65].

Another factor studied in obese asthmatics was L-citrulline supplemented through 2 weeks in a dose of 15 g/d. In a pilot study in poorly controlled obese asthmatics, the authors showed an increase in FeNO and, at the same time, an improvement in ACQ [66].

As noted by Brown et al., not only the type of intervention used—diet and/or exercise—but also the method of proposing it to the patient may affect the achievement of the effect. For younger children, it is important to involve the parent in the therapeutic process, while adolescents can be offered age-appropriate methods—the use of games and applications for mobile devices that support the treatment process [67]. The role of doctor-patient communication is also emphasized. A study conducted by Alexander et al. showed that obese teenagers with asthma want to hear from their doctor about possible weight-loss interventions to help them better control their asthma. The patients underlined that they would like such a conversation to be started by a doctor, and what is more, they also wanted the parents’ participation [68].

## 5. Conclusions

Asthma and obesity seem to be associated, and the relationship between these two diseases is certainly multifactorial and based on genetic, immunological, and mechanical aspects. Diet is a compelling modifiable factor in obesity and asthma prevention and control. It is crucial to recommend appropriate lifestyle modifications to children with excess body weight—encouraging them to change eating habits and increase physical exertion. In obese children with asthma, the above recommendations must be supplemented with optimal drug therapy and control of the course of both diseases. There are still some unresolved questions about the possibility of asthma prevention and improving asthma control by diet modifications in obese children. For example, should a specific diet be dedicated to different phenotypes of asthma? Is loss of weight through exercise a more sufficient form of prevention than diet intervention, and does the time of intervention matter? More research on this topic is needed. Future studies concerning the effect of weight loss and exercise on asthma control may provide a better understanding of the complex relationships between asthma and obesity and may increase the effectiveness of therapeutic processes.

## References

[1] Matricardi P.M., Gruber C., Wahn U., Lau S. (2007). The asthma-obesity link in childhood: Open questions, complex evidence, a few answers only. Clin. Exp. Allergy.

[2] Rzehak P., Wijga A.H., Keil T., Eller E., Bindslev-Jensen C., Smit H.A., Weyler J., Dom S., Sunyer J., Mendez M. (2013). Body mass index trajectory classes and incident asthma in childhood: Results from 8 European Birth Cohorts–A Global Allergy and Asthma European Network initiative. J. Allergy Clin. Immunol..

[3] Lang D.M., Butz A.M., Duggan A.K., Serwint J.R. (2004). Physical activity in urban schoolaged children with asthma. Pediatrics.

[4] Okubo Y., Nochioka K., Hataya H., Sakakibara H., Terakawa T., Testa M. (2016). Burden of obesity on pediatric in patients with acute asthma exacerbation in the United States. J. Allergy Clin. Immunol. Pract..

[5] Holguin F., Bleecker E.R., Busse W.W., Calhoun W.J., Castro M., Erzurum S.C., Fitzpatrick A.M., Gaston B., Israel E., Jarjour N.N. (2011). Obesity and asthma: An association modified by age of asthma onset. J. Allergy Clin. Immunol..

[6] Diaz J., Farzan S. (2014). Clinical implications of the obese-asthma phenotypes. Immunol. Allergy Clin. N. Am..

[7] Ricketts H.C., Cowan D.C. (2019). Asthma, obesity and targeted interventions: An update. Curr. Opin. Allergy Clin. Immunol..

[8] Dixon A.E., Pratley R.E., Forgione P.M., Kaminsky D.A., Whittaker-Leclair L.A., Griffes L.A., Garudathri J., Raymond D., Poynter M.E., Bunn J.Y. (2011). Effects of obesity and bariatric surgery on airway hyperresponsiveness, asthma control, and inflammation. J. Allergy Clin. Immunol..

[9] Peters U., Dixon A., Forno E. (2018). Obesity and Asthma. J. Allergy Clin. Immunol..

[10] Scott H.A., Gibson P.G., Garg M.L., Wood L.G. (2011). Airway inflammation is augmented by obesity and fatty acids in asthma. Eur. Respir. J..

[11] Forno E., Young O.M., Kumar R., Simhan H., Celedón C. (2014). Maternal obesity in pregnancy, gestational weight gain, and risk of childhood asthma. Pediatrics.

[12] Brüske I., Flexeder C., Heinrich J. (2014). Body mass index and the incidence of asthma in children. Curr. Opin. Allergy Clin. Immunol..

[13] Sithole F., Douwes J., Burstyn I. (2008). Body Mass Index and Childhood Asthma: A Linear Association?. J. Asthma.

[14] Gilliland F.D., Berhane K., Islam T., McConnell R., Gauderman W.J., Gilliland S.S., Avol E., Peters J.M. (2003). Obesity and the Risk of Newly Diagnosed Asthma in School-age Children. Am. J. Epidemiol..

[15] Azizpour Y., Delpisheh A., Montazeri Z. (2018). Effect of childhood BMI on asthma: A systematic review and meta-analysis of case-control studies. BMC Pediatr..

[16] Forno E., Lescher R., Strunk R., Weiss S., Fuhlbrigge A., Celedon J.C., Childhood Asthma Management Program Research Group (2011). Decreased response to inhaled steroids in overweight and obese asthmatic children. J. Allergy Clin. Immunol..

[17] Boulet L.P., Franssen E. (2007). Influence of obesity on response to fluticasone with or without salmeterol in moderate asthma. Respir. Med..

[18] Melen E., Himes B.E., Brehm J.M., Boutaoui N., Klanderman B.J., Sylvia J.S., Lasky-Su J. (2010). Analyses of shared genetic factors between asthma and obesity in children. J. Allergy Clin. Immunol..

[19] Zhu Z., Guo Y., Shi H., Liu C.L., Panganiban R.A., Chung W., O’Connor L.J., Himes B.E., Gazal S., Hasegawa K. (2020). Shared genetic and experimental links between obesity-related traits and asthma subtypes in UK Biobank. J. Allergy Clin. Immunol..

[20] Thomsen S.F., Ulrik C.S., Kyvik K.O., Sørensen T.I., Posthuma D., Skadhauge L.R., Steffensen I., Backer V. (2007). Association between obesity and asthma in a twin cohort. Allergy.

[21] Hallstrand T.S., Fischer M.E., Wurfel M.M., Afari N., Buchwald D., Goldberg J. (2005). Genetic pleiotropy between asthma and obesity in a community-based sample of twins. J. Allergy Clin. Immunol..

[22] Dixon A.E., Peters U. (2018). The effect of obesity on lung function. Expert. Rev. Respir. Med..

[23] Shore S.A. (2010). Obesity, airway hyperresponsiveness, and inflammation. J. Appl. Physiol..

[24] Huang F., Del-Río-Navarro B.E., Torres-Alcántara S., Pérez-Ontiveros J.A., Ruiz-Bedolla E., Saucedo-Ramírez O.J., Villafaña S., Sánchez Muñoz F., Bravo G., Hong E. (2017). Adipokines, asymmetrical dimethylarginine, and pulmonary function in adolescents with asthma and obesity. J. Asthma.

[25] Johnston R.A., Theman T.A., Lu F.L., Terry R.D., Williams E.S., Shore S.A. (2008). Diet-induced obesity causes innate airway hyperresponsiveness to methacholine and enhances ozone-induced pulmonary inflammation. J. Appl. Physiol..

[26] Nasiri Kalmarzi R., Ataee P., Mansori M., Moradi G., Ahmadi S., Kaviani Z., Khalafi B., Kooti W. (2017). Serum levels of adiponectin and leptin in asthmatic patients and its relation with asthma severity, lung function and BMI. Allergol. Immunopathol..

[27] Fantuzzi G. (2005). Adipose tissue, adipokines, inflammation. J. Allergy Clin. Immunol..

[28] Quek Y.W., Sun H.L., Ng Y.Y., Lee H.S., Yang S.F., Ku M.S., Lu K.H., Sheu J.N., Lue K.H. (2010). Associations of serum leptin with atopic asthma and allergic rhinitis in children. Am. J. Rhinol. Allergy.

[29] Guler N., Kirerleri E., Ones U., Tamay Z., Salmayenli N., Darendeliler F. (2004). Leptin: Does it have any role in childhood asthma?. J. Allergy Clin. Immunol..

[30] Medoff B.D., Okamoto Y., Leyton P., Weng M., Sandall B.P., Raher M.J., Kihara S., Bloch K.D., Libby P., Luster A.D. (2009). Adiponectin-deficiency increases allergic airway inflammation and pulmonary vascular remodeling. Am. J. Respir. Cell Mol. Biol..

[31] Baek H.S., Kim Y.D., Shin J.H., Kim J.H., Oh J.W., Lee H.B. (2011). Serum leptin and adiponectin levels correlate with exercise-induced bronchoconstriction in children with asthma. Ann. Allergy Asthma Immunol..

[32] Dooley A.A., Pillai D.K. (2021). Paediatric Obesity-Related Asthma: Disease Burden and Effects on Pulmonary Physiology. Paediatr. Respir. Rev..

[33] Forno E., Han Y.Y., Mullen J., Celedón J.C. (2018). Overweight, Obesity, and Lung Function in Children and Adults-A Meta-analysis. J. Allergy Clin. Immunol. Pract..

[34] Forno E., Weiner D.J., Mullen J., Sawicki G., Kurland G., Han Y.Y., Cloutier M.M., Canino G., Weiss S.T., Litonjua A.A. (2017). Obesity and Airway Dysanapsis in Children with and without Asthma. Am. J. Respir. Crit. Care Med..

[35] Eijkemans M., Mommers M., Draaisma J.M., Thijs C., Prins M.H. (2012). Physical activity and asthma: A systematic review and meta-analysis. PLoS ONE.

[36] Jensen M.E., Gibson P.G., Collins C.E., Hilton J.M., Wood L.G. (2013). Diet-induced weight loss in obese children with asthma: A randomized controlled trial. Clin. Exp. Allergy.

[37] Luna-Pech J.A., Torres-Mendoza B.M., Luna-Pech J.A., Garcia-Cobas C.Y., Navarrete-Navarro S., Elizalde-Lozano A.M. (2014). Normocaloric diet improves asthma-related quality of life in obese pubertal adolescents. Int. Arch. Allergy Immunol..

[38] Abd El-Kader M.S., Al-Jiffri O., Ashmawy E.M. (2013). Impact of weight loss on markers of systemic inflammation in obese Saudi children with asthma. Afr. Health Sci..

[39] Willeboordse M., van de Kant K.D.G., Tan F.E., Mulkens S., Schellings J., Crijns Y., van der Ploeg L., van Schayck C.P., Dompeling E. (2016). A Multifactorial Weight Reduction Programme for Children with Overweight and Asthma: A Randomized Controlled Trial. PLoS ONE.

[40] Ma J., Strub P., Xiao L., Lavori P.W., Camargo C.A., Wilson S.R., Gardner C.D., Buist A.S., Haskell W.L., Lv N. (2015). Behavioral weight loss and physical activity intervention in obese adults with asthma. A randomized trial. Ann. Am. Thorac. Soc..

[41] Scott H.A., Gibson P.G., Garg M.L., Pretto J.J., Morgan P.J., Callister R., Wood L.G. (2013). Dietary restriction and exercise improve airway inflammation and clinical outcomes in overweight and obese asthma: A randomized trial. Clin. Exp. Allergy.

[42] Johnson O., Gerald L.B., Harvey J., Roy G., Hazucha H., Large C., Burke A., McCormack M., Wise R.A., Holbrook J.T. (2022). An Online Weight Loss Intervention for People With Obesity and Poorly Controlled Asthma. J. Allergy Clin. Immunol. Pract..

[43] Freitas P.D., Ferreira P.G., Silva A.G., Stelmach R., Carvalho-Pinto R.M., Fernandes F.L., Mancini M.C., Sato M.N., Martins M.A., Carvalho C.R. (2017). The Role of Exercise in a Weight-Loss Program on Clinical Control in Obese Adults with Asthma. A Randomized Controlled Trial. Am. J. Respir. Crit. Care Med..

[44] Dias-Júnior S.A., Reis M., de Carvalho-Pinto R.M., Stelmach R., Halpern A., Cukier A. (2014). Effects of weight loss on asthma control in obese patients with severe asthma. Eur. Respir. J..

[45] Santos L.M., Ramos B., Almeida J., Loureiro C.C., Cordeiro C.R. (2019). The impact of weight loss beyond lung function: Benefit with respect to asthma outcomes. Pulmonology.

[46] Sugerman H.J., Sugerman E.L., DeMaria E.J., Kellum J.M., Kennedy C., Mowery Y., Wolfe L.G. (2003). Bariatric surgery for severely obese adolescents. J. Gastrointest. Surg..

[47] Zeki A.A., Oldham J., Wilson M., Fortenko O., Goyal V., Last M., Last A., Patel A., Last J.A., Kenyon N.J. (2013). Statin use and asthma control in patients with severe asthma. BMJ Open.

[48] Dogaru C.M., Nyffenegger D., Pescatore A.M., Spycher B.D., Kuehni C.E. (2014). Breastfeeding and childhood asthma: Systematic review and meta-analysis. Am. J. Epidemiol..

[49] Wallby T., Lagerberg D., Magnusson M. (2017). Relationship Between Breastfeeding and Early Childhood Obesity: Results of a Prospective Longitudinal Study from Birth to 4 Years. Breastfeed Med..

[50] Wasilewska E., Małgorzewicz S., Szczepankiewicz A., Myśliwczyk D., Hennig M., Jassem E., Skotnicka M. (2022). Are obesity and asthma in school-age children still strongly related to breastfeeding in infancy?—A real-life study. Eur. Rev. Med. Pharmacol. Sci..

[51] Berthon B.S., Macdonald-Wicks L.K., Gibson P.G., Wood L.G. (2013). Investigation of the association between dietary intake, disease severity and airway inflammation in asthma. Respirology.

[52] Wood L.G., Shivappa N., Berthon B.S., Gibson P.G., Hebert J.R. (2015). Dietary inflammatory index is related to asthma risk, lung function and systemic inflammation in asthma. Clin. Exp. Allergy.

[53] Garcia-Marcos L., Canflanca I.M., Garrido J.B., Varela A.L., Garcia-Hernandez G., Guillen-Grima F., Gonzalez-Diaz C., Carvajal-Urueña I., Arnedo-Pena A., Busquets-Monge R.M.A. (2007). Relationship of asthma and rhinoconjunctivitis with obesity, exercise and Mediterranean diet in Spanish schoolchildren. Thorax.

[54] Amazouz H., Roda C., Beydon N., Lezmi G., Bourgoin-Heck M., Just J., Momas I., Rancière F. (2021). Mediterranean diet and lung function, sensitization, and asthma at school age: The PARIS cohort. Pediatr. Allergy Immunol..

[55] Zhang Y., Lin J., Fu W., Liu S., Gong C., Dai J. (2019). Mediterranean diet during pregnancy and childhood for asthma in children: A systematic review and meta-analysis of observational studies. Pediatr. Pulmonol..

[56] Sexton P., Black P., Metcalf P., Wall C.R., Ley S., Wu L., Sommerville F., Brodie S., Kolbe J. (2013). Influence of mediterranean diet on asthma symptoms, lung function, and systemic inflammation: A randomized controlled trial. J. Asthma.

[57] Bseikri M., McCann J.C., Lal A., Fong E., Graves K., Goldrich A., Block D., Gildengoren G.L., Mietus-Snyder M., Shigenaga M. (2018). A novel nutritional intervention improves lung function in overweight/obese adolescents with poorly controlled asthma: The Supplemental Nutrition in Asthma Control (SNAC) pilot study. FASEB J..

[58] Turer C.B., Lin H., Flores G. (2013). Prevalence of vitamin D deficiency among overweight and obese US children. Pediatrics.

[59] O’Sullivan B.P., James L., Majure J.M., Bickel S., Phan L.T., Serrano Gonzalez M., Staples H., Tam-Williams J., Lang J., Snowden J. (2021). Obesity-related asthma in children: A role for vitamin D. Pediatr. Pulmonol..

[60] Peterson C. (2015). Vitamin D deficiency and childhood obesity: Interactions, implications, and recommendations. Nutr. Diet. Suppl..

[61] Mirzakhani H., Al-Garawi A., Weiss S.T., Litonjua A.A. (2015). Vitamin D and the development of allergic disease: How important is it?. Clin. Exp. Allergy J. Br. Soc. Allergy Clin. Immunol..

[62] Wortsman J., Matsuoka L.Y., Chen T.C., Lu Z., Holick M.F. (2000). Decreased bioavailability of vitamin D in obesity. Am. J. Clin. Nutr..

[63] Drincic A.T., Armas L.A., Van Diest E.E., Heaney R.P. (2012). Volumetric dilution, rather than sequestration best explains the low vitamin D status of obesity. Obesity.

[64] Pugliese G., Liccardi A., Graziadio C., Barrea L., Muscogiuri G., Colao A. (2022). Obesity and infectious diseases: Pathophysiology and epidemiology of a double pandemic condition. Int. J. Obes..

[65] Lang J.E., Mougey E.B., Hossain M.J., Livingston F., Balagopal P.B., Langdon S., Lima J.J. (2019). Fish Oil Supplementation in Overweight/Obese Patients with Uncontrolled Asthma. A Randomized Trial. Ann. Am. Thorac. Soc..

[66] Holguin F., Grasemann H., Sharma S., Winnica D., Wasil K., Smith V., Cruse M.H., Perez N., Coleman E., Scialla T.J. (2019). L-Citrulline increases nitric oxide and improves control in obese asthmatics. JCI Insight.

[67] Brown T., Moore T.H., Hooper L., Gao Y., Zayegh A., Ijaz S., Elwenspoek M., Foxen S.C., Magee L., O’Malley C. (2019). Interventions for preventing obesity in children. Cochrane Database Syst. Rev..

[68] Alexander G.L., Olden H.A., Troy T., Miree C.A., Joseph C.L.M. (2018). Overweight adolescents and asthma: Revealing motivations and challenges with adolescent-provider communication. J. Asthma.

